# Semiquantitative Evaluation of Muscle Repair by Diffusion Tensor Imaging in Mice

**DOI:** 10.1002/jbm4.10040

**Published:** 2018-03-05

**Authors:** Junichi Hata, Sakiko Mizuno, Yawara Haga, Masayuki Shimoda, Yae Kanai, Kazuhiro Chiba, Hideyuki Okano, Masaya Nakamura, Keisuke Horiuchi

**Affiliations:** ^1^ Department of Physiology Keio University School of Medicine Tokyo Japan; ^2^ Central Institute for Experimental Animals Kanagawa Japan; ^3^ Department of Orthopedic Surgery Keio University School of Medicine Tokyo Japan; ^4^ Department of Orthopedics Tokyo Dental College Ichikawa General Hospital Ichikawa City Chiba Japan; ^5^ Department of Radiological Sciences Tokyo Metropolitan University Tokyo Japan; ^6^ Department of Pathology Keio University School of Medicine Tokyo Japan; ^7^ Department of Orthopedic Surgery National Defense Medical College Saitama Japan

**Keywords:** MAGNETIC RESONANCE IMAGING, DIFFUSION TENSOR IMAGING, MUSCLE INJURY, MUSCLE REPAIR, ADAM10

## Abstract

Muscle injury is one of the most common traumas in orthopedic and sports medicine. However, there are only a few treatment options with marginal clinical benefits for this condition. Muscle repair after injury involves multiple and complex processes, such as the inflammation phase, regeneration phase, and remodeling phase. To develop a treatment modality and to examine the efficacy of novel interventions and agents for patients with muscle injuries, it is essential to establish a reliable and sensitive method to monitor the changes in muscle structure and status during muscle repair. Diffusion‐weighted magnetic resonance imaging has been widely used to assess the diffusivity of water molecules in tissue. When it is used in combination with diffusion tensor imaging (DTI), the microstructure of muscle tissue can be indirectly depicted. In the present study, we evaluated the time‐course changes in the diffusivity and anisotropy in muscles by DTI and histology after injury in mice. We found that the diffusivity and anisotropy exhibit distinct kinetics during muscle repair and that these kinetics were significantly altered in mutant mice with a defect in muscle regeneration. Our data show that muscle repair processes can be readily evaluated and monitored by DTI technique and suggest that DTI can be clinically applied for assessing muscle injury and repair in humans. © 2018 The Authors. *JBMR Plus* is published by Wiley Periodicals, Inc. on behalf of American Society for Bone and Mineral Research.

## Introduction

Muscle injuries, such as muscle strain, muscle pull, and muscle tear, are among the most common orthopedic and sports traumas. In severe cases, surgical repair of the damaged muscles may be performed. Meanwhile, there are currently no effective conservative treatments other than immobilization and the administration of anti‐inflammatory drugs.^1^, ^2^ In establishing a treatment modality for muscle injuries, it is crucial to develop a reliable and sensible method to evaluate the regenerative and healing processes of muscle injuries. Muscle injuries and the accompanying edema are often evaluated by conventional T1‐weighted and T2‐weighted magnetic resonance imaging (MRI).^3^, ^4^ However, there is no established diagnostic modality to evaluate the healing processes of muscle injuries. Therefore, there is a great demand for a technique to monitor and quantify muscle repair.

Diffusion‐weighted imaging (DWI) is an MRI‐based method that has been used to evaluate the diffusivity of water molecules in tissue.^5^, ^6^, ^7^ Because the diffusivity of water molecules is constantly affected by the surrounding microenvironment, DWI allows for the indirect assessment of the microstructure of tissue. Diffusion tensor imaging (DTI) is a technique by which the degree of anisotropy of water molecules can be deduced based on the DWI datasets.^6^, ^7^ Because neurons usually have long axons and thus exhibit a highly anisotropic structure, the clinical use of DTI has been best explored in the field of neurology.^5^, ^8^, ^9^, ^10^, ^11^, ^12^ Accordingly, the diagnostic value of DTI has been investigated for various neurological disorders, including acute stroke, brain tumors, multiple sclerosis, traumatic brain and spinal injuries, and neuropsychiatric disorders, to name a few examples.^5^ Of note, DTI is also increasingly applied to study the structure and physiology of skeletal muscle.^13^, ^14^ Skeletal muscle consists of numerous muscle fibers that are highly anisotropic in structure. This unique structural property of skeletal muscle permits the use of DTI to quantify the directional anisotropy and thereby to detect subtle changes in muscle structure and status.

In the present study, we aimed to clarify the applicability and usefulness of DTI in evaluating muscle injury and repair using a cardiotoxin‐induced muscle injury model in mice. The anisotropy and diffusivity of water molecules were evaluated at different time points after injury with the corresponding histology. In addition, we also used mutant mice that are devoid of muscle satellite cells and exhibit a severely compromised muscle regeneration capacity to test if our method is sensitive enough to depict the defects in the mutant mice.^(15)^ Our results illustrate the distinct kinetics of diffusivity during muscle repair in mice and suggest that DTI is a valid tool for evaluating muscle injury and regeneration in humans.

## Materials and Methods

### Mice

Eight‐week‐old to 9‐week‐old mice were used in the present study. The generation of *Adam10^flox/flox^* mice was previously described.^16^ *Adam10^flox/flox^* mice exhibit no apparent defect and used as wild‐type control animals in the present study (henceforth referred to as wild‐type mice).^15^ *Adam10^Pax7^* mice were generated through crossing *Adam10^flox/flox^* mice with *Pax7^tm2.1(cre/ERT2)Fan^*/J transgenic mice.^15^, ^17^ Conditional excision of the floxed allele was done by intraperitoneal injection of tamoxifen (75 µg/kg; Toronto Research Chemicals, Toronto, ON, Canada) dissolved in corn oil (20 mg/mL), as described.^15^ ADAM10 is a membrane‐bound protease that is essential for processing Notch.^15^, ^16^, ^18^ Notch is critically involved in the maintenance of the quiescence of satellite cells (muscle‐specific stem cells that are indispensable for muscle regeneration) and loss of the Notch signaling results in the induction of satellite cell differentiation.^19^, ^20^, ^21^ In a previous study, we showed that conditional abrogation of ADAM10 activity under the control of a *Pax7* promoter leads to a depletion of satellite cell pool and a severe defect in muscle regeneration.^15^ These mutant mice show no apparent anomalies under unchallenged conditions; however, they are incapable of regenerating muscle fibers after injury and the damaged muscle tissues are ultimately replaced by adipose and fibrous tissues. All mice were maintained under specific‐pathogen‐free conditions and were given food (Clea Rodent Diet CE‐2; Clea Japan, Tokyo, Japan) and water *ad libitum*. Mice were group housed (three to five mice per cage). All animal experiments were approved by the Institutional Animal Care and Use Committee of the Keio University School of Medicine (approval number: 11022).

### Muscle injury model

Muscle injury was induced by intramuscular injection of 50 µL cardiotoxin/phosphate‐buffered saline (10 µM) in the tibialis anterior (TA) muscle.^15^ All procedures were performed under general anesthesia with an intraperitoneal injection of a mixture of sterilized water, medetomidine (30 µg/mL), midazolam (400 µg/mL), and butorphanol (500 µg/mL) at a volume of 0.1 mL/10 g body weight. The mice were closely monitored until fully recovered from the anesthesia. The mice were euthanized at 3, 14, and 28 days postinjury (DPI). The hind limbs were collected and fixed with 4% paraformaldehyde for MRI and histological analyses.

### Histology

The fixed tissues were embedded in paraffin and sectioned in parallel and perpendicular to the orientation of muscle fibers. Sections were stained with H&E. Images were acquired using an Olympus FSX100 fluorescence microscope (Olympus, Tokyo, Japan) and Olympus FSX‐BSW software, and processed using Adobe Photoshop CS6 (Adobe, San Jose, CA, USA).

### MRIs

A 7‐T MRI scanner (Bruker Biospin, Ettlingen, Germany) and an inner diameter of 22 mm volume coil (Bruker Biospin, Ettlingen, Germany) were used for MRIs, as described.^12^, ^22^ The maximum magnetic field gradient amplitude was 700 mT/m. For the DTI pulse sequence, we used the pulse gradient stimulated echo pulse sequence.^23^ This method enables investigators to obtain strong signals even in tissues with extremely short T2 values (eg, hepatic parenchyma [40 ms], skeletal muscle [50 ms], and cardiac muscle [60 ms]).^24^, ^25^ The imaging parameters used in the present study were as follows: repetition time/echo time/mixing time = 2000/15.9/48.6 ms; δ/Δ = 2.1/50.7 ms; b‐value = 800 s/mm^2^; average = 2; field of view = 12.8 × 8.4 mm^2^; matrix size = 128 × 84; number of slices = 20; slice thickness = 0.6 mm with no gap; motion probing gradient moment = six directions (xy, xz, yz, −xy, −xz, −yz); and total scanning time = 26 min 36 s. During the imaging procedure, the specimens were wrapped in a sponge and soaked in a fluorine solution (Sumitomo 3M Limited, Tokyo, Japan).

### DTI processing

**Figure 1:**
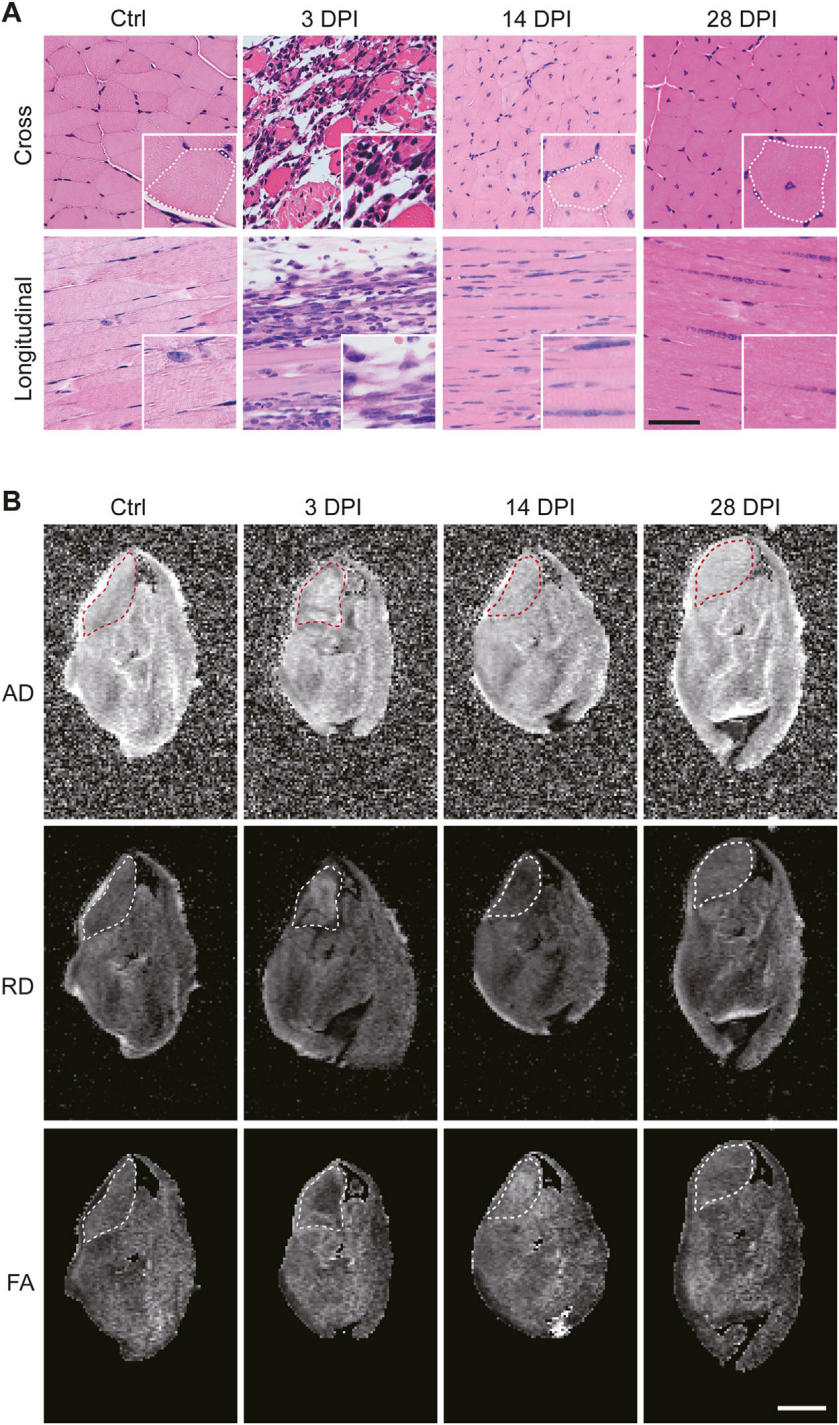
Sections and scalar maps of the TA muscle after muscle injury. (*A*) Cross‐sections and longitudinal sections of the Ctrl hind limb and the hind limbs collected at 3, 14, and 28 DPI. The insets show a magnified image. The dotted lines delineate a muscle fiber. Scale bar = 100 µm for the low magnification and 50 µm for the insets. (*B*) AD, RD, and FA maps of the TA muscle. The dotted lines delineate the TA muscle (ROI). Scale bar = 2 mm.

DTI indices were computed using the Diffusion Toolkit (developed by Drs. Ruopeng Wang and Van J. Wedeen, Martinos Center for Biomedical Imaging, Massachusetts General Hospital; http://trackvis.org/). The fractional anisotropy (FA), axial diffusivity (AD), and radial diffusivity (RD) values are evaluated at different time points after injury. AD reflects the linear diffusivity paralleled to the muscle fiber orientation. RD reflects the linear diffusivity perpendicular to the muscle fiber orientation and is positively correlated with the muscle fiber diameter. FA reflects the degree of anisotropic diffusion and ranges from 0 (represents isotropic diffusion) to 1 (represents anisotropic diffusion).^(13)^ AD was deduced from the principal eigenvalue (λ1). RD was calculated by the mean of the second and third eigenvalues ([λ2 + λ3]/2). FA was calculated using the equation below.

$$FA=\sqrt{\frac{3}{2}}\sqrt{\frac{{\left(\lambda 1-D\right)}^{2}+{\left(\lambda 2-D\right)}^{2}+{\left(\lambda 3-D\right)}^{2}}{{\lambda 1}^{2}+{\lambda 2}^{2}+{\lambda 3}^{2}}}$$

where D = (λ1 + λ2 + λ3)/3. The TA muscles in the imaged lower leg were targeted for measurement; the regions of interest (ROIs) were carefully selected on MRIs (the dotted lines in Figs. 1 *B* and 3*B*). Each ROI was applied to the calculated DTI images (FA, λ1, λ2, λ3) to measure the indices. Image J software version 1.37 (NIH, Bethesda, MD, USA; https://imagej.nih.gov/ij/) was used for the selection and measurement of the ROI.

### Statistical analysis

One sample Student's *t* test (hypothetical value = 1) was used to determine statistical significance. A value of *p* < 0.05 was considered statistically significant. GraphPad Prism software (version 6.05; La Jolla, CA, USA) was used for statistical calculations. The data are presented as the mean ± SD.

## Results

To evaluate the time‐course changes in diffusivity and anisotropy during muscle repair, we analyzed the TA muscles at three different time points (3, 14, and 28 DPI) after cardiotoxin injection. The contralateral limbs were used as controls (Ctrl). As shown in Fig. 1 *A*, the sections of the muscles at 3 DPI showed degradation of muscle fibers, robust infiltration of immune cells, and the formation of edema, reflecting severe inflammation. By 14 DPI, the inflammation was fully resolved and the regeneration of muscle fibers was observed. However, the cross‐sectional area of the muscle fibers remained smaller than that of Ctrl muscle fibers. Additionally, most of the muscle fibers still had central nuclei, indicating that the muscle fibers were still immature and not fully functional at this time point. The sections of 28 DPI specimens showed that the thickness of the muscle fibers was comparable to those in Ctrl and that there was a marked decrease in the number of fibers with central nuclei compared with the 14‐DPI specimens, suggesting that the damaged muscle tissues were almost fully recovered by 28 DPI. These observations confirm that the specimens collected from 3, 14, and 28 DPI represent the degeneration/inflammation phase, the regeneration phase, and the completion of the remodeling phase, respectively.

**Figure 2:**
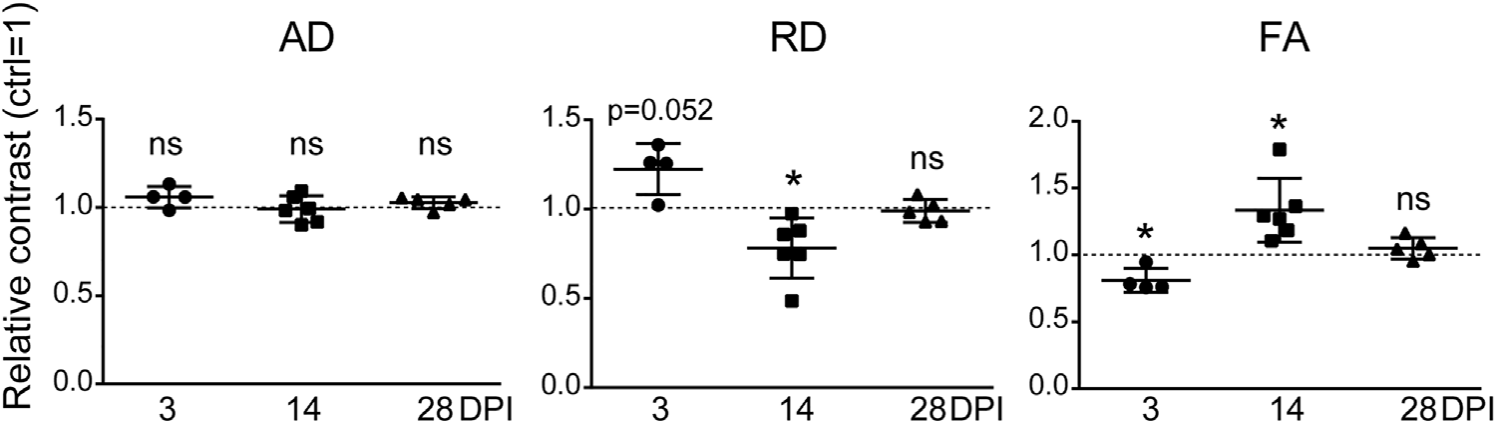
Time‐course changes in the AD, RD, and FA values during muscle repair. The average of each parameter in the uninjured TA muscle is set to 1 (dotted line). *n* = 4 mice (3 DPI), 6 mice (14 DPI), and 5 mice (28 DPI). *p < 0.05. ns = not significant.

The specimens collected at the designated time points were subjected to MRI analysis. The DWI data were acquired and processed to obtain AD, RD, and FA maps, as described in the Materials and Methods. Figure 1 *B* shows representative images of the scalar maps (AD, RD, and FA maps) of a Ctrl specimen and the hind limbs collected at 3, 14, and 28 DPI. The ROI was manually set to cover the cross‐sectional area of the TA muscle (the dotted lines in Fig. 1 *B*). The ROI value of the Ctrl specimens was set to 1 to calculate the relative contrast. Figure 2 shows the time‐course changes in the relative contrast of AD, RD, and FA maps of the TA muscle after the induction of injury. The AD value remained nearly static through the course, showing no significant changes in the value at each time point. On the other hand, the RD value temporally elevated at 3 DPI, decreased at 14 DPI, and recovered to the basal level by 28 DPI (approximately 18.6% increase in the cross‐sectional area of the muscle fibers from 14 DPI to 28 DPI; data not shown). The FA value appeared to behave in a nearly opposite manner to the RD value: it decreased at 3 DPI, elevated at 14 DPI, and recovered to the basal level by 28 DPI. Taken together, these observations show that the anisotropy of muscle tissue decreases at the inflammation phase, temporally increases at the regeneration phase, and recovers to the basal level by the completion of remodeling phase, and that the kinetics in the anisotropy is primarily derived from the changes in the RD values.

**Figure 3:**
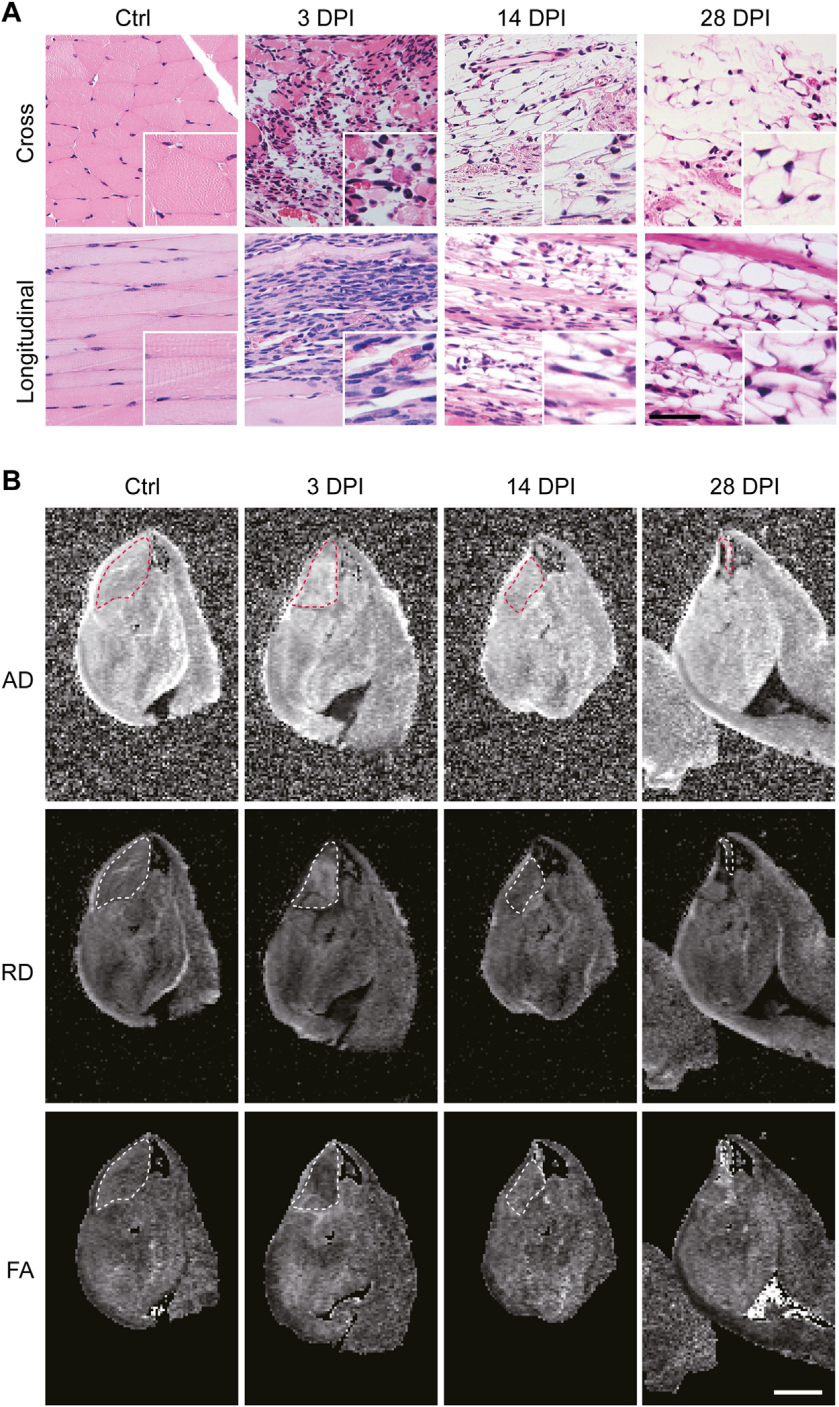
Sections and scalar maps of the TA muscle of *Adam10^Pax7^* mice after muscle injury. (*A*) Cross‐sections and longitudinal sections of the Ctrl hind limb and the hind limbs collected at 3, 14, and 28 DPI. The insets show a magnified image. Scale bar = 100 µm for the low magnification and 50 µm for the insets. (*B*) AD, RD, and FA maps of the TA muscle. The dotted lines delineate the TA muscle (ROI). Scale bar = 2 mm.

We next aimed to validate the present method using *Adam10^Pax7^* mice, in which satellite cells can be fully depleted by multiple tamoxifen treatments.^15^ Using this animal model, we exactly repeated the experiments and analyses. In accordance to the previous study,^15^ muscle regeneration was severely impaired in *Adam10^Pax7^* mice (Fig. 3 *A*). The sections at 3 DPI appeared nearly identical to those of wild‐type mice (Fig. 1 *A*), which are characterized by expanded interstitial space and massive infiltration of immune cells. However, at 14 DPI, there were very few regenerating muscle fibers but numerous immature adipocytes and fibroblasts. By 28 DPI, the damaged tissues were entirely replaced by mature adipocytes and fibrous tissues with only a few remaining muscle fibers. These specimens were subjected to DWI acquisition and DTI analysis under the same protocol used for wild‐type mice. Figure 3 *B* shows representative images of the scalar maps of a Ctrl specimen and the hind limbs collected at 3, 14, and 28 DPI from *Adam10^Pax7^* mice. The ROI was set to delineate the cross‐sectional area of the TA muscle (the dotted lines in Fig. 3 *B*).

**Figure 4:**
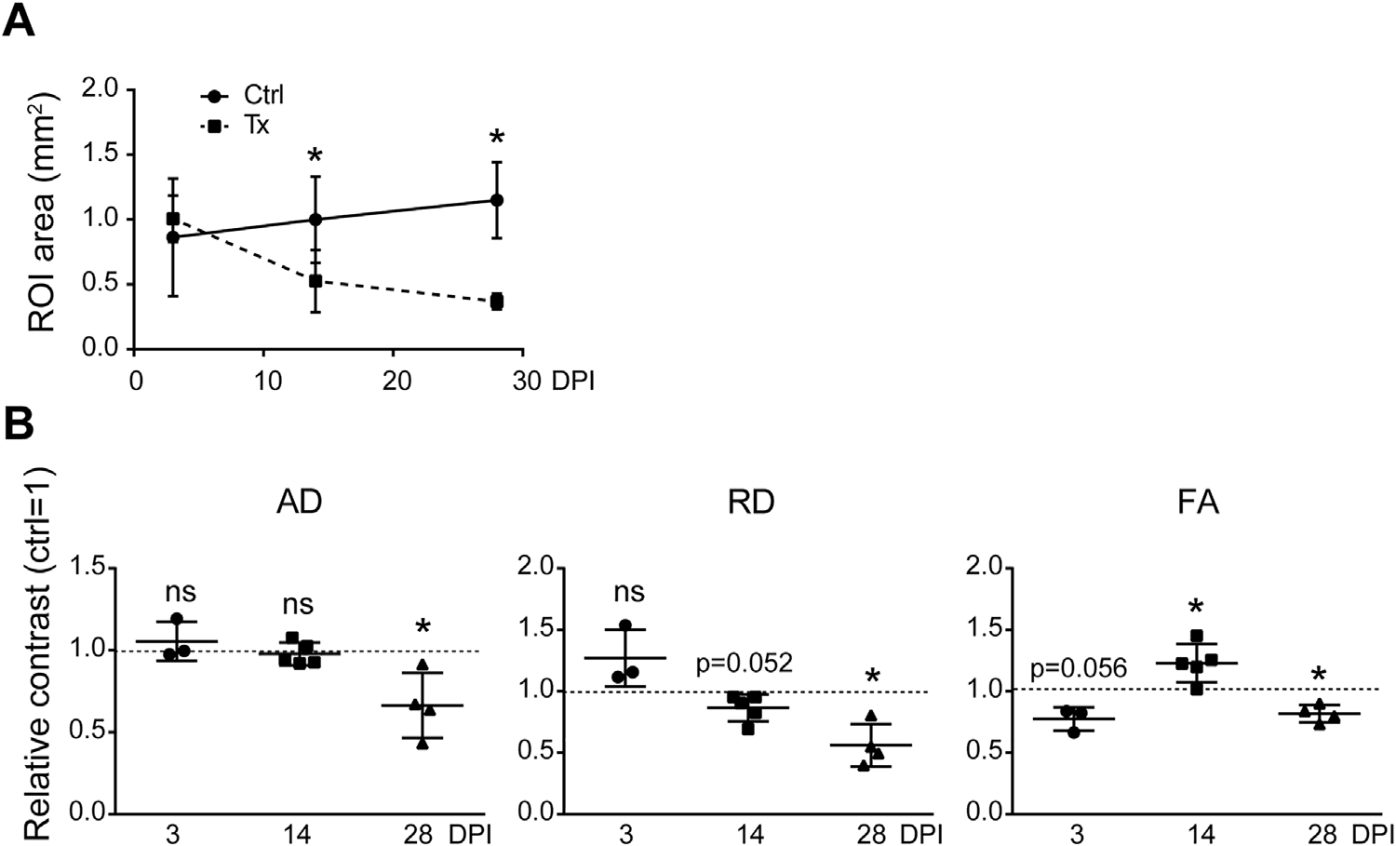
(*A*) Time course changes of the ROI area after cardiotoxin injection in *Adam10^Pax7^* mice. *p < 0.05. (*B*) Time‐course changes in the AD, RD, and FA values during muscle healing in *Adam10^Pax7^* mice. The average of each parameter in the uninjured TA muscle is set to 1 (dotted line). *n* = 3 mice (3 DPI), 5 mice (14 DPI), and 4 mice (28 DPI). *p < 0.05. ns = not significant; Tx = cardiotoxin‐treated limb.

As expected, there was a significant decrease in the ROI area in the cardiotoxin‐treated limbs compared to the contralateral control limbs at 14 and 28 DPI owing to impaired muscle regeneration (Fig. 4 *A*). There were no marked changes in the FA values at 3 and 14 DPI compared with the Ctrl (Fig. 4 *B*); however, unlike in wild‐type mice (Fig. 2), there was a significant decrease in the FA values at 28 DPI. In a similar vein, the RD values showed an increase at 3 DPI and a decrease at 14 DPI as observed in wild‐type mice. However, the RD value further lowered and did not recover to the basal level at 28 DPI. The FA values in *Adam10^Pax7^* mice significantly decreased at 28 DPI, whereas they showed a similar trend to those in wild‐type mice at 3 and 14 DPI. These results showed that the defect in muscle regeneration leads to a decrease in both the AD and RD values, as well as the anisotropy (FA), at the completion of the remodeling phase, and suggest that the present method is sensitive enough to depict the defect in muscle repair in *Adam10^Pax7^* mice.

## Discussion

The present study illustrates the time‐course changes in the diffusivity and anisotropy of water molecules in muscle tissues during muscle repair in mice. Our data indicate that the FA value decreases at the inflammation phase, is elevated during the generation phase, and recovers to the basal level as the muscle fibers become mature. Our data also suggest that the kinetics in the FA value is predominantly derived from the changes in the RD value and that the AD value remains nearly static after muscle injury and during muscle repair, at least under the present experimental settings. In addition, we also found that *Adam10^Pax7^* mice, a mutant line with severely impaired muscle regeneration capacity, exhibited altered kinetics in both diffusivity and anisotropy, supporting the validity of the present method.

**Figure 5:**
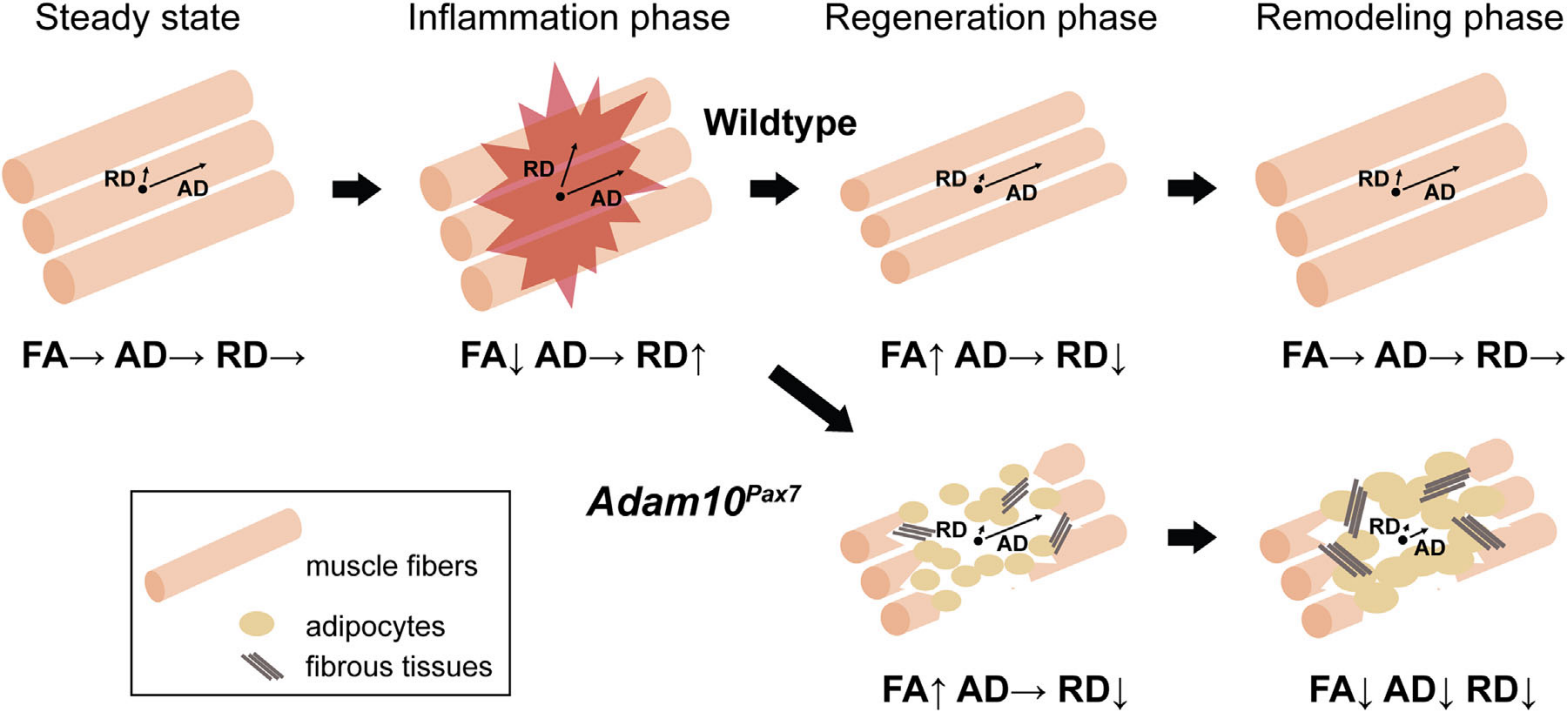
Proposed model summarizing the results of the present study. Please see the Discussion for details.

Given the nature of DWI, which can only depict the average diffusivity of water molecules in a given area, movement of water molecules can only be indirectly interpreted. In addition, because muscle tissues are not only composed of muscle fibers, but also of nerves, blood vessels, and extracellular matrix, the DWI datasets in the present study does not solely reflect the diffusivity of water molecules in muscle fibers. Nevertheless, we would like to propose a putative model based on our observations (Fig. 5). Under the steady‐state conditions, the diffusivity of water molecules is restricted to the direction parallel to the orientation of muscle fibers, leading to anisotropic diffusion (high FA). Muscle injury, which is accompanied by the damages to the cytoplasmic membrane and basal membrane of muscle fibers, results in the loss of the anisotropic structure of muscle fibers and formation of edema (leading to lower anisotropy). As a result, the diffusivity of water molecules in the direction perpendicular to the orientation of muscle fibers will increase (leading to higher RD). On the other hand, because the loss of the fiber structure does not affect AD, the AD value will remain relatively static. Once the regeneration of muscle fibers is achieved, the anisotropic structure will also be recovered. However, because muscle fibers are immature and thinner at this stage, RD will be more restricted than in the steady state (leading to lower RD and higher FA). Afterward, RD will increase during the remodeling phase until the muscle fibers become fully mature. In *Adam10^Pax7^* mice, the regeneration and remodeling of muscle fibers are severely compromised and the damaged area is replaced with adipocytes and fibrous tissues (Fig. 3 *A*). Because adipose and fibrous tissues hamper the diffusivity of water molecules, both RD and AD will ultimately be highly restricted, resulting in lower FA.

Several studies have shown that tissue damage, including muscle injuries, often results in an elevated diffusivity of water molecules and a decrease in anisotropy.^26^, ^27^, ^28^, ^29^, ^30^, ^31^, ^32^ Because injury can potentially disrupt the microstructure of any given tissue and result in the formation of edema, it is not surprising that the diffusivity of water molecules becomes less restricted after tissue damage. Our results that the FA value decreased at the inflammation phase are in accordance with this notion. On the other hand, studies exploring the potential use of DWI in evaluating muscle repair are relatively few.^28^, ^29^, ^30^, ^33^ Of note, Esposito and colleagues^29^ also investigated the time‐course changes in the FA value in mice and showed similar results to ours regarding the kinetics of the FA value (a sharp decrease after injury, then an increase, and a gentle decline during remodeling). Although there are several differences in the study designs, their results are consistent with our findings and further support our proposed model (Fig. 5).

There are several limitations to the present study. Most critically, although the muscle injury model used in the present study is widely accepted as a tool to study muscle regeneration in vivo, there are very few published studies on histological analysis of muscle repair in humans. In this regard, it is not clear to what extent this mouse model will recapitulate the muscle healing processes in humans. In a similar vein, it is also unlikely that *Adam10^Pax7^* mice mimic a particular human disease. Nevertheless, the results of the present and past studies^15^ indicate that the *Adam10^Pax7^* mice may serve as a model to study how a decreased number or activity of satellite cells, as often found in aged people and patients with muscular dystrophy, would impact muscle repair. Additionally, to avoid the noises derived from body movements, we did not use live animals for the acquisition of DWI. Therefore, the potential effects of blood flow on DWI during muscle regeneration remain to be elucidated. Further studies, especially in human subjects, are necessary to address these issues and to understand the usefulness of DTI in evaluating muscle regeneration in humans.

In conclusion, our data clearly show the kinematic changes in the diffusivity and anisotropy of water molecules after muscle injury and muscle repair with the corresponding histology. Additionally, our study may be the first to show how the DTI parameters will be affected in muscles devoid of satellite cells after injury. Although the results of the present study need to be cautiously interpreted, we believe that the present study further underscores the validity of DTI in evaluating muscle repair processes and may provide a basis for clinical application.

## Disclosures

All authors state that they have no conflict of interest.
